# Epigenetic modifications in cardiac fibrosis: recent evidence of new pharmacological targets

**DOI:** 10.3389/fmolb.2025.1583446

**Published:** 2025-05-02

**Authors:** Marian Pérez, Mónica Gómez, Jairo Castellar-López, Patricio Araos, Evelyn Mendoza-Torres, Samir Bolívar

**Affiliations:** ^1^ Molecular Pharmacology Laboratory, Pharmacy and Pharmacology Research Group, Universidad del Atlántico, Barranquilla, Colombia; ^2^ Faculty of Health, Exact and Natural Sciences, Universidad Libre de Colombia, Seccional Barranquilla, Barranquilla, Colombia; ^3^ Faculty of Health Sciences, Universidad de la Guajira, Riohacha, Colombia; ^4^ Institute of Biomedical Sciences, Universidad Autonoma de Chile, Santiago de Chile, Chile

**Keywords:** cardiac fibrosis, cardiac fibroblasts, epigenetic modifications, cardiovascular epigenetics, non-coding RNA, DNA methylation

## Abstract

Cardiac fibrosis (CF) is characterized by the excessive deposition of collagen types I (COI I) and III (COI III), primarily mediated by cardiac fibroblasts (CFB). Recent advances in epigenetic research have enhanced our understanding of the molecular mechanisms underlying CF and have facilitated the identification of novel therapeutic strategies targeting key proteins and signaling pathways involved in its progression. Epigenetic modifications, including DNA methylation, histone modifications, and non-coding RNAs (ncRNAs), are structural and chemical alterations that regulate gene expression and cellular responses without changing the DNA sequence. Investigating the role of epigenetic enzymes in CF may reveal promising pharmacological targets. This review summarizes current evidence on epigenetic modifications implicated in CF and discusses their potential as therapeutic targets for modulating this pathological process.

## 1 Introduction

Epigenetics refers to heritable changes in gene expression that are not caused by alterations in the DNA sequence but rather by chemical modifications at the DNA level, such as DNA methylation and histone modifications. These modifications influence DNA accessibility and chromatin structure, thereby regulating gene expression, cell differentiation, X-chromosome inactivation, and genomic stability (Loscalzo and Handy, 2014; Cao et al., 2014; Ling and Rönn, 2019). Chromatin is composed of DNA and histones, which undergo various modifications, including phosphorylation, methylation, acetylation, propionylation, and acylation. Additionally, DNA and RNA can be chemically modified through methylation to regulate gene expression (Sun et al., 2022).

Epitranscriptomic regulation refers to post-transcriptional chemical modifications in coding and ncRNAs that modulate their function and contribute to cellular homeostasis (Orsolic et al., 2023). RNA in all living organisms can undergo over 100 distinct post-transcriptional modifications, among which N6-methyladenosine (m6A) is the most abundant internal modification in eukaryotic mRNA. This modification influences mRNA metabolism and translation, directing transcripts toward different cellular fates by promoting their processing, stability, or degradation (Zhao et al., 2017).

Post-translational modifications (PTMs) occur on specific amino acid residues within the regulatory domains of target proteins, influencing their stability and function. These regulatory regions, known as degrons, are modulated by PTMs that act as molecular signals, either accelerating protein degradation (PTM-activated degrons) or inhibiting degradation to stabilize proteins (PTM-inactivated degrons) (Lee et al., 2023). Among the most extensively studied PTMs are histone modifications. Given that chromatin serves as the template for all DNA-mediated processes, histone modifications play a crucial role in regulating chromatin structure and function. For example, a combination of H4K8 acetylation, H3K14 acetylation, and H3S10 phosphorylation is associated with transcriptional activation, whereas H3K9 trimethylation and the absence of H3 and H4 acetylation correlate with transcriptional repression (Peterson and Laniel, 2004).

The reversible nature of these chemical modifications makes epigenetic regulators, proteins responsible for “writing,” “erasing,” and “reading” the epigenetic code, attractive therapeutic targets (Esteller, 2017). This potential has been increasingly explored in the context of cardiovascular diseases, including CF, a condition characterized by excessive extracellular matrix (ECM) deposition in the myocardium following cardiac injury (Xu et al., 2022). The development and progression of CF involve complex molecular and cellular mechanisms, in which epigenetic regulation plays a fundamental role in both physiological and pathological cardiac remodeling (Aguado-Alvaro et al., 2024; Liu et al., 2023).

In CF, epigenetic dysregulation contributes to maladaptive remodeling by promoting excessive ECM accumulation and CFBs activation. Alterations in DNA methylation and histone acetylation have been implicated in these processes. Notably, histone deacetylase (HDAC) inhibitors have demonstrated anti-fibrotic effects by reducing inflammation and cardiac hypertrophy, thereby attenuating fibrosis-associated remodeling (Loscalzo and Handy, 2014). Additionally, the transition of CFBs into myoFIBs, a hallmark of fibrosis, has been linked to changes in DNA methylation. Given the central role of histone deacetylation in maladaptive remodeling, HDAC inhibitors are being explored as potential therapeutic agents (Loscalzo and Handy, 2014; Cao et al., 2014).

Recent evidence indicates that epigenetic modifications affecting both DNA and RNA are critically involved in CF progression by regulating profibrotic biomarkers and modulating CFBs behavior, activation, and differentiation (see Table 1) (Li et al., 2021; Mathiyalagan et al., 2019; Dong et al., 2023). Understanding these mechanisms may open new avenues for therapeutic interventions targeting epigenetic regulators to mitigate fibrosis-related cardiac dysfunction.

**TABLE 1:** Synthesis of experimental studies exploring the role of epigenetically active proteins in CF.

Epigenetic modifications				
Protein	Epigenetic - function	Experimental model of cardiac fibrosis	Findings	References
METTL3	m6A	Murine model of myocardial infarction	Increased expression of COL I and III, α-SMA, and enhanced proliferation and differentiation of CFBs	Li et al. (2021)
METTL3	m6A	Murine model of CF	METTL3 methylates GAS5, promoting its degradation and activating mitochondrial fission via Drp1, ultimately leading to the proliferation and migration of CFBs	Tu et al. (2023)
METTL3	m6A	Murine model of myocardial infarction	METTL3 methylates Drp1, activates mitochondrial fission, and promotes CF.	Huang et al. (2023)
METTL3	m6A	METTL3 floxed murine model/Cardiomyocyte-specific Mettl3 knockout murine model	Inhibition of METTL3 can alleviate myocardial fibrosis and inflammation, and prevent cardiomyocyte death under reperfusion injury conditions by disrupting DNA-PKcs/Fis1-dependent mitochondrial fission	Ma et al. (2024)
METTL3	m6A	Murine model of myocardial infarction	METTL3 increased the m^6^A levels of TNC mRNA and enhanced its stability, thereby contributing to the regulation of myocardial dysfunction	Cheng et al. (2023)
METTL3	m6A	Murine model of CF	Silencing METTL3 downregulates IGFBP3 expression and inhibits cardiac fibroblast (CFB) activation and the progression of CF.	Ding et al. (2023a)
METTL3	m6A	Murine model of myocardial infarction	MetBil interacts with METTL3 and regulates the expression of methylated fibrosis-associated genes	Zhuang et al. (2023)
METTL3	m6A	Murine model of cardiac remodeling	Deficiencies in METTL3 and METTL14 significantly attenuated Ang II–induced myocardial inflammation and fibrosis by reducing m^6^A modifications on MyD88 and TGF-β1 mRNAs. This reduction inhibited NF-κB pathway activation and decreased the expression of CXCR2 and TGF-β1	Li et al. (2024a)
FTO	m6A	Male and female murine (C57BL/6) and rats (Sprague-Dawley rats)	Selective demethylation of cardiac contractile transcripts, including SERCA2a, MYH6, MYH7, and RyR2	Mathiyalagan et al. (2019)
FTO	m6A	Neonatal rat CFBs	LE prevents the downregulation of FTO and reduces cardiac fibroblast proliferation, migration, and collagen synthesis	Meng et al. (2024)
FTO	m6A	Myocardial infarction Sprague-Dawley rats	HIF-1α binds to the promoter region of FTO and suppresses its expression. Functionally, FTO inhibits collagen synthesis	Wang et al. (2024)
WTAP	m6A	Murine model of diabetic CF.	Androgen receptor (AR) directly interacts with the mitochondrial lipid oxidation enzyme Decr1. Overexpression of AR suppresses Decr1-mediated mitochondrial lipid oxidation, thereby inhibiting CFBs proliferation and migration	Song et al. (2023)
*Alkbh5*	m6A	Leptin receptor-deficient mice cardiac fibroblast-specific *Notch 1* conditional knockout mice Cre mice	ALKBH5- and YTHDF2-mediated degradation of Notch1 promotes mitochondrial fission in diabetic cardiac fibrosis	Liu et al. (2024)
Dot1L	Histone modification	Adult Sprague–Dawley rats and neonatal rat	The interaction between Dot1L and FOX3 promotes the activation of CFBs	Xu et al. (2022)
PRMT5	Histone modification	Murine model of CF.	Activation and differentiation of cardiac fibroblasts (CFBs) are mediated through the PRMT5/Smad3 interaction	Dong et al., 2023
PRMT5/MLL1	Histone modification	Murine models with transverse aortic constriction (TAC)	PRMT5-induced arginine methylation and MLL1-mediated lysine methylation initiate α-SMA gene transcription, thereby promoting myoFIBs differentiation	Katanasaka et al. (2024)
p300	Histone modification	Murine model of CF	p300 interacts with SMAD2, as inhibition of p300 activity prevents TGF-β1–induced SMAD2 phosphorylation and nuclear translocation	Lim et al. (2021)
p300	Histone modification	Murine model of hypertension	MyoFIBs differentiation	Rai et al. (2019)
KDM5B	Histone modification	Murine models with transverse aortic constriction (TAC)	KDM5B promotes the binding of CFs to ATF3, an antifibrotic regulator, thereby inhibiting its expression	Wang et al. (2022)
KDM5B	Histone modification	Murine models with transverse aortic constriction (TAC)	KDM5B-mediated activation of cardiac fibroblasts (CFBs) is associated with the activation of the Wnt/β-catenin signaling pathway	Tang et al. (2022)
HDAC8	Histone modification	Experimental murine model of heart failure	Increased MMP12	Zhou et al. (2024)
BRD4	Histone modification	Murine model of pathological cardiac remodeling	BRD4 is functionally associated with the TGF-β1/Smad2/3 signaling pathway	He et al. (2022)
BRD4	Histone modification	Neonatal Sprague-Dawley rats	BRD4 downregulation is associated with the inhibition of Ang II–induced transdifferentiation of CFBs	Han et al. (2023)
DNMT1	DNA methylation	Murine model of CF	Inhibition of DNMT1 leads to the overexpression of miR-152-3p, which suppresses the proliferation of CFBs	Xu et al. (2021)
DNMT3A	DNA methylation	Murine model of CF	Hypermethylation of the sFRP3 promoter is associated with CFBs activation and CF.	Jiang et al. (2024)
miR-223	miRNA	Experimental murine model of heart failure	miR-223 enhances proliferation, migration, and differentiation of CFBs, thereby promoting CF, partially through the regulation of RASA1	Liu et al. (2018)
miRNA-99b-3p	miRNA	Murine model of CF	miRNA-99b-3p interacts with GSK3β, leading to Smad3 activation and promoting CF.	Yu et al. (2021)
miRNA-331	miRNA	Murine model of CF	Interaction with the TGF-β/Smad3 pathway inhibits profibrotic gene expression in MyoFIBs	Yousefi et al. (2021)
LncRNA GAS5	LncRNA	Murine model of CF	Interaction with the NLRP3 inflammasome reduces the expression of caspase-1 and IL-1β, thereby alleviatingCF.	Zhang et al. (2024)
CircNSD1	circRNA	Murine model of myocardial infarction	CFBs proliferation is promoted through activation of the Wnt/β-catenin signaling pathway	Ji et al. (2024)

This section describes the proteins analyzed and their epigenetic functions (such as histone modifications, DNA methylation, and non-coding RNAs), along with the experimental models of fibrosis used, and the observed effects on CFBs activation, ECM remodeling, and the progression of the fibrotic phenotype.

## 2 Cardiac fibrosis: classification and cellular mechanisms

CF can be classified into focal scar fibrosis and diffuse fibrosis, depending on its etiology and distribution. Focal scar fibrosis, also known as reparative fibrosis, replaces dead cardiomyocytes following myocardial infarction or other ischemic events. In contrast, diffuse fibrosis, or reactive fibrosis, occurs in the interstitial and perivascular spaces without significant cardiomyocyte loss and is more commonly associated with heart failure (Xu et al., 2022; Heymans et al., 2015). The primary distinction between these two forms of fibrosis lies in their origin. In clinical practice, CF often presents as a combination of both: following myocardial infarction, reparative fibrosis develops within the necrotic zone, while reactive fibrosis occurs in the surrounding border zone, driven by an inflammatory response triggered by cell necrosis. This process significantly impacts cardiac function. While replacement fibrosis is generally considered irreversible and essential for maintaining structural integrity, reactive fibrosis is regarded as maladaptive and contributes to pathological remodeling (Delacroix and Hulot, 2022).

### 2.1 The role of cardiac fibroblasts in fibrosis progression

**FIGURE 1:**
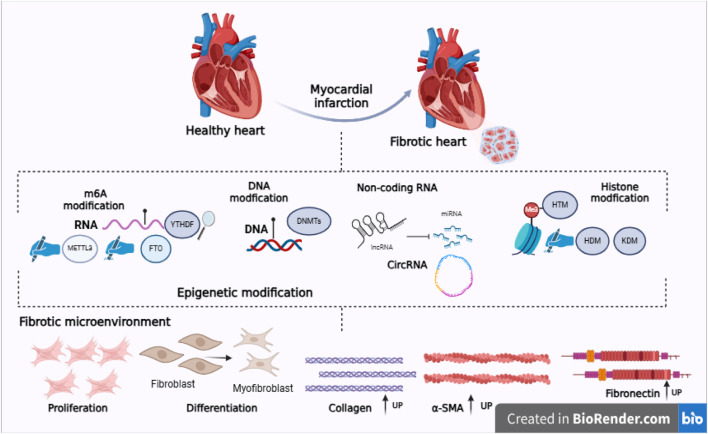
In response to cardiac injury, CFBs become activated, proliferate, and differentiate into cardiac myofibroblasts (myoFIBs). This phenotypic transition is associated with the upregulation of fibrotic markers, including COI I, COI III, alpha-smooth muscle actin (α-SMA), and fibronectin. Accumulating evidence indicates that various epigenetic modifications play a key role in regulating the activation, proliferation, and fibrogenic behavior of CFBs, as well as the expression of proteins that characterize the fibrotic cardiac phenotype.

CFBs are the primary cellular mediators of the fibrotic process. CFBs, in general, generate and maintain structurally diverse ECM-rich connective tissues that provide mechanical resilience, facilitate elastic recovery, and support organ function. In addition to ECM synthesis, CFBs contribute to tissue homeostasis by influencing the microarchitecture, biomechanics, and biochemical composition of the ECM. They also regulate cellular communication through the secretion of soluble mediators, including cytokines, growth factors, and metabolites. Following myocardial injury, CFBs become activated and differentiate into myoFIBs, a highly proliferative and secretory phenotype characterized by excessive ECM deposition. This transition plays a central role in fibrotic remodeling and contributes to the pathological stiffening of the myocardium (Liu et al., 2021; Plikus et al., 2021; Tallquist, 2020) (see Figure 1).

### 2.2 Molecular signaling and therapeutic targets

In CFBs, transforming growth factor beta (TGF-β) acts as a central effector in the fibrotic response, as it strongly induces collagen synthesis and promotes the differentiation of CFBs into myoFIBs. In addition, TGF-β facilitates the acquisition of a cellular phenotype oriented toward ECM preservation, characterized by increased expression of antiproteases and the secretion of enzymes involved in collagen cross-linking (Frangogiannis, 2020).

Moreover, CFBs express a wide array of receptors that regulate their activation, proliferation, and ECM production. Among these, β_2_-adrenergic receptors (β_2_AR) modulate fibroblast function through the activation of protein kinase A (PKA) and the exchange protein directly activated by cAMP (EPAC), which differentially influence the behavior of both CFBs and myoFIBs. Additionally, angiotensin II (Ang II) receptors particularly the AT_1_R subtype stimulate collagen synthesis and fibroblast proliferation; however, their overexpression may lead to apoptosis. CFBs also express bradykinin B_1_ and B_2_ receptors, whose activation triggers signaling pathways that inhibit collagen synthesis and secretion through nitric oxide- and prostacyclin I_2_ (PGI_2_)-dependent mechanisms. Beyond their structural role, CFBs actively participate in the inflammatory response during cardiac repair. They express several immune-associated receptors, including Toll-like receptor 4 (TLR4), NLRP3, and the interferon receptor (IFNAR), all of which have attracted increasing interest as potential therapeutic targets for modulating fibrotic responses in CF (Díaz-Araya et al., 2015).

## 3 m^6^A modification in mRNA

m^6^A is the most prevalent internal modification in eukaryotic messenger RNA and plays a pivotal role in regulating RNA transcription, splicing, stability, degradation, and translation (Ye et al., 2023). This dynamic and reversible modification is mediated by three groups of proteins commonly referred to as “writers,” “readers,” and “erasers,” which together orchestrate the m^6^A epitranscriptomic landscape (Oerum et al., 2021).

### 3.1 Writers: Installation of m^6^A marks

The core methyltransferase complex responsible for m^6^A deposition comprises methyltransferase-like 3 (METTL3), methyltransferase-like 14 (METTL14), and Wilms tumor 1-associated protein (WTAP). METTL3 serves as the catalytic subunit, transferring a methyl group from S-adenosylmethionine (SAM) to specific adenosine residues within the RNA sequence. METTL14 acts as an RNA-binding scaffold, enhancing substrate recognition and stabilizing the METTL3-METTL14 complex. WTAP, although catalytically inactive, functions as a regulatory component that guides the complex to specific RNA targets and may also interact with other RNA species and associated regulatory proteins (Tan et al., 2024).

A homolog of METTL3, methyltransferase-like 16 (METTL16), has been identified as a regulator of intracellular SAM levels and a methyltransferase for specific small nuclear RNAs (snRNAs), expanding the functional scope of the m^6^A machinery (Sun et al., 2019).

### 3.2 Readers: interpreting the m^6^A signal

Reader proteins selectively bind to m^6^A-modified transcripts, mediating downstream processes such as translation enhancement, transcript degradation, and subcellular localization. The YTH domain family proteins—including YTHDF1, YTHDF2, YTHDF3, YTHDC1, and YTHDC2—are the best-characterized m^6^A readers. In addition, members of the heterogeneous nuclear ribonucleoprotein (HNRNP) family, such as HNRNPA2/B1, HNRNPC, and HNRNPG, can recognize m^6^A-modified RNAs and influence splicing or RNA structure.

Furthermore, insulin-like growth factor 2 mRNA-binding proteins (IGF2BP1/2/3) have been shown to bind m^6^A-containing mRNAs and promote their stability and translation in an m^6^A-dependent manner (Gao et al., 2024).

### 3.3 Erasers: removal of m^6^A marks

The reversibility of m^6^A modification is controlled by demethylases, also known as “erasers.” The fat mass and obesity-associated protein (FTO) demethylates m^6^A by oxidizing it to N6-hydroxymethyladenosine and N6-formyladenosine, which are subsequently hydrolyzed to adenine. ALKBH5 (AlkB homolog 5) is another demethylase that removes m^6^A in a similar manner, thereby fine-tuning the transcriptome in response to physiological or pathological stimuli (Hu et al., 2025).

### 3.4 Additional regulators of the m^6^A machinery

Other accessory proteins contribute to the regulation and specificity of the m^6^A methylation machinery. RNA-binding motif protein 15 and 15B (RBM15/RBM15B) are involved in recruiting the methyltransferase complex to target RNAs. Zinc finger CCCH domain-containing protein 13 (ZC3H13) facilitates RNA interaction with the complex, while VIRMA (VIR-like m^6^A methyltransferase-associated) protein enhances complex stability and localization at specific transcript regions (Gao et al., 2024; Hu et al., 2025).

## 4 Role of M6A IN CF

### 4.1 METTL3 and CF

Recent studies have increasingly highlighted the role of m6A RNA modification in heart failure, particularly its association with METTL3. One of the earliest reports demonstrated elevated METTL3 levels in fibrotic cardiac tissue from mice following myocardial infarction, as well as in cultured CFBs stimulated with TGF-β1. METTL3 overexpression promoted CFBs proliferation, differentiation into myoFIBs, and collagen deposition, whereas its silencing had the opposite effect. The proposed mechanism involves activation of the classical TGF-β1/SMAD2/3 signaling pathway, as METTL3 upregulates SMAD2/SMAD3 expression (Li et al., 2021).

In addition to its role in fibrotic signaling, METTL3 has been implicated in metabolic reprogramming, particularly through glycolysis modulation. METTL3 methylates androgen receptor (AR) mRNA, promoting its degradation via a YTHDF2-dependent mechanism. This downregulation of AR enhances hypoxia-inducible factor 1-alpha (HIF-1α) signaling, a key glycolytic pathway, thereby stimulating CFBs proliferation (Zhou et al., 2022).

Furthermore, METTL3 regulates mitochondrial fission, a process closely linked to CF. Elevated METTL3 expression promotes mitochondrial fragmentation, increasing CFBs proliferation and migration. Conversely, METTL3 downregulation attenuates these effects. Mechanistically, METTL3 mediates m6A methylation of the long non-coding RNA (LncRNA) GAS5, which interacts with dynamin-related protein 1 (Drp1), a key regulator of mitochondrial fission. Overexpression of GAS5 suppresses Drp1 activity, mitigating fibrosis progression (Tu et al., 2023).

A recent study introduced a novel therapeutic approach using a timed-release microneedle system containing S-adenosylhomocysteine (SAH), an inhibitor of the METTL3-METTL14 complex. In experimental models of TGF-β1-induced and hypoxia-ischemic cardiac injury, inhibition of METTL3-METTL14 reduced mitochondrial fragmentation and myoFIBs transdifferentiation. This treatment significantly improved cardiac function and decreased fibrosis markers (collagen I, collagen III, and α-SMA) in rat models. Mechanistically, the inhibitor suppressed Drp1 expression and phosphorylation, preserving mitochondrial integrity and reducing cardiomyocyte loss post-ischemic injury (Huang et al., 2023).

Additional research has linked METTL3 to ischemia/reperfusion (I/R)-induced CF through mitochondrial fission. In METTL3 knockout mice, reduced cardiac remodeling and fibrosis were observed compared to wild-type controls. METTL3 expression correlated positively with caspase-3 activity, implicating it in cardiomyocyte apoptosis. The study proposed that METTL3 regulates mitochondrial fission via DNA-dependent protein kinase catalytic subunit (DNA-PKcs), which phosphorylates mitochondrial fission protein 1 (Fis1) (Ma et al., 2024).

Other studies have explored METTL3’s regulation of ECM components. Tenascin-C (TNC), a glycoprotein involved in cardiomyocyte proliferation and apoptosis, is stabilized by METTL3-mediated m6A methylation, exacerbating myocardial dysfunction (Cheng et al., 2023). Similarly, METTL3 influences CFBs activity by modulating insulin-like growth factor-binding protein 3 (IGFBP3), which is positively associated with CF. METTL3 silencing reduces IGFBP3 expression, attenuating pathological remodeling (Ding et al., 2023a).

Recent findings have also identified the LncRNA, MetBil as a regulator of METTL3 stability. Elevated MetBil levels correlate with increased expression of fibrosis markers (COI I and COI III) and CFBs proliferation. Mechanistically, MetBil inhibits METTL3 ubiquitination, stabilizing its protein levels and enhancing its activity (Zhuang et al., 2023).

Finally, METTL3 inhibition using STM2457, delivered via erythrocyte-derived microvesicles, has shown promise in models of cardiac device-associated fibrosis. In patients with ventricular septal defects who developed conduction blocks after occluder implantation, METTL3 and METTL14 were upregulated in peripheral blood mononuclear cells. The inhibition of METTL3/14 decreased m6A methylation of myeloid differentiation primary response protein 88 (MyD88) and TGF-β1 mRNAs, thereby suppressing the activation of the nuclear factor kappa-light-chain-enhancer of activated B cells (NF-κB) signaling pathway and downregulating CXC motif chemokine receptor 2 (CXCR2) and TGF-β1 expression. Consequently, monocyte infiltration, inflammation, and CF were significantly reduced (Li J. et al., 2024).

### 4.2 FTO and m6A modification in CF

FTO plays a key role in CF through its m6A demethylase activity. Following cardiac injury, FTO expression is significantly downregulated, contributing to adverse cardiac remodeling. FTO’s cardioprotective effects have been attributed to its selective demethylation of transcripts encoding cardiac contractile proteins, including sarcoplasmic/endoplasmic reticulum Ca^2+^ ATPase 2a (SERCA2a), myosin heavy chains (MYH6/7), and ryanodine receptor 2 (RyR2). This demethylation enhances mRNA stability and protein expression, thereby supporting contractile function (Mathiyalagan et al., 2019).

The antifibrotic role of FTO has also been demonstrated *in vitro* using leonurine (LE), a natural cardioprotective compound. LE inhibited Ang II-induced CFBs proliferation, migration, and collagen synthesis. Ang II suppresses FTO expression, whereas LE restores it. Silencing FTO reversed the effects of LE, promoting fibrosis by increasing collagen I, collagen III, and α-SMA expression. These findings suggest that FTO-mediated m6A demethylation is essential for LE’s antifibrotic action (Meng et al., 2024).

Additionally, FTO has been shown to suppress collagen synthesis after myocardial infarction. One study identified IGF2BP3 as a stabilizer of profibrotic mRNAs, including glutamyl-prolyl-tRNA synthetase (EPRS). FTO’s demethylase activity antagonizes this process by destabilizing these mRNAs, reducing fibrosis progression (Wang et al., 2024).

### 4.3 Other components of the m6A machinery in cardiac fibrosis

Recent studies have identified WTAP as a novel regulator of CF. WTAP promotes mitochondrial lipid oxidation, facilitating CFBs activation and differentiation into myoFIBs. This effect appears to be mediated by WTAP’s m6A-dependent downregulation of AR mRNA (Song et al., 2023).

Additionally, Notch homolog 1 (Notch1) has been implicated in fibrosis regulation. Reduced Notch1 expression correlates with increased m6A methylation and mitochondrial fission. Specific deletion of Notch1 in CFBs exacerbates CF in diabetic mouse models. ALKBH5, an m6A demethylase, mediates Notch1 mRNA demethylation at its 3′untranslated region (3′UTR), influencing mRNA stability and fibrotic remodeling (Liu et al., 2024).

The role of m6A RNA modification extends beyond CF, having been implicated in cancer progression (Luo et al., 2018; Wang et al., 2020; Zhu et al., 2024) and fibrotic diseases (Xiao et al., 2024; Tsai et al., 2024). Growing evidence suggests that m6A methylation is a key regulator of CF, particularly through METTL3 and FTO. The opposing roles of these enzymes highlight their potential as therapeutic targets. Further research into the molecular consequences of METTL3 and FTO modulation will be critical for developing epigenetic therapies to mitigate CF and adverse remodeling.

## 5 Histone modification

In multicellular eukaryotic organisms, all cells generally contain the same genome, which is packaged within the nucleus into a highly complex and dynamic superstructure known as chromatin. The fundamental unit of chromatin is the nucleosome, composed of DNA wrapped around an octamer of core histones—H2A, H2B, H3, and H4, forming approximately two superhelical turns. This structure is further stabilized by the linker histone H1, which facilitates chromatin compaction. Extending from the nucleosome core, histone tails are highly conserved peptide sequences subject to numerous PTMs that play pivotal roles in regulating chromatin structure and function (Fellows and Varga-Weisz, 2020).

Chromatin exists in a highly dynamic state, transitioning between transcriptionally active and repressive conformations. Histone PTMs serve as key determinants of these chromatin states and influence essential biological processes, including transcription, DNA replication, repair, and remodeling (Izzo and Schneider, 2010). Among the most extensively studied PTMs are acetylation, phosphorylation, and methylation of specific histone residues (Rothbart and Strahl, 2014).

Histone acetylation typically occurs at the ε-amino groups of lysine residues in the N-terminal tails of histones H3 and H4 and is generally associated with transcriptional activation by promoting chromatin relaxation. In contrast, histone lysine methylation is a more intricate regulatory mechanism, with its functional outcome depending on the specific lysine residue modified and the degree of methylation. For instance, trimethylation of histone H3 at lysine 4 (H3K4me3) is linked to transcriptional activation, whereas trimethylation at lysine 9 (H3K9me3) or lysine 27 (H3K27me3) is typically associated with transcriptional repression and heterochromatin formation (Fellows and Varga-Weisz, 2020).

Histone methylation plays a critical role in cardiac remodeling and is among the most predominant PTMs in this context. This modification is catalyzed by histone methyltransferases (HMTs), which transfer methyl groups from the donor coenzyme SAM to specific lysine or arginine residues on histone tails, thereby modulating chromatin structure and gene expression (Miller and Grant, 2013). HMTs are categorized into three major families: SET domain-containing enzymes (responsible for the majority of lysine methylation, also known as KMTs), DOT1-like histone lysine methyltransferases (DOT1L), which specifically methylate H3K79, and PRMTs, which catalyze arginine methylation. Conversely, histone demethylases (HDMs) counteract these modifications by removing methyl groups, thereby maintaining a dynamic regulatory balance. HDMs are classified into two major groups: amine oxidases and Jumonji C (JmjC) domain-containing dioxygenases, the latter being iron- and α-ketoglutarate-dependent (Black et al., 2012; Gong and Miller, 2019).

Methylated histones function as docking sites for various reader proteins containing methyl-binding domains that interpret these modifications. These reader domains include ankyrin, BAH, DCD, MBT, TTD, and zf-CW, among others (Musselman et al., 2012) (See Table 2).

**TABLE 2:** Histone-methylation enzymes.

Methylation									
Writers	KMTs	KMT1A KMT1B KMT1C KMT1D KMT1E KMT1F	KMT2A KMT2B KMT2C KMT2D KMT2E KMT2F KMT2G KMT2H	KMT3A KMT3B KMT3C KMT3D KMT3E KMT3F KMT3G	KMT4	KMT5A KMT5B KMT5C	KMT6	KMT7	KMT8A KMT8B KMT8C KMT8D KMT8E KMT8F
	PRMTs	PRMT1 PRMT2 PRMT3 PRMT4 PRMT5 PRMT6 PRMT7 PRMT8 PRMT9							
Eraser	KDMs	KDM1A KDM1B	KDM2A KDM2B	KDM3A KDM3B KDM3C	KDM4A KDM4B KDM4C KDM4D	KDM5A KDM5B KDM5C KDM5D	KDM6A KDM6B	KDM7A KDM7B KDM7C	KDM8
Readers	BAH Ankyrin DCD MBT TTD zf-CW								

Mammalian histone methyltransferases (including KMT and PRMT) and demethylases (KDM) are classified according to their ability to transfer or reverse one, two, or three methyl groups. Abbreviations: KMT: histone lysine methyltransferases; PRMT: protein arginine methyltransferases; KDM: histone demethylases; BAH: bromo-adjacent homology; DCD: chromobarrel, chromodomain, double chromodomain; MBT: malignant brain tumor; TTD: Tudor tandem domain; zf-CW: zinc finger CW.

Histone acetylation is catalyzed by HATs, which transfer an acetyl group from acetyl-coenzyme A to the ε-amino group of lysine residues on histone tails. This modification neutralizes the positive charge of lysine, weakening histone, DNA interactions and promoting a more relaxed chromatin structure that facilitates transcription. HATs are classified into several families, including GCN5, p300/CBP, and the MYST family, which includes MOZ, Ybf2/Sas3, Sas2, and Tip60 (Yao et al., 2024; Miziak et al., 2024; Guo et al., 2018).

Histone acetylation is reversible and tightly regulated by HDACs. These enzymes are categorized based on their catalytic mechanisms into two main groups: canonical zinc-dependent HDACs and SIRTs, which are NAD + -dependent deacetylases (Park and Kim, 2020; Poziello et al., 2021).

Acetylated histones are recognized by specific reader domains that interpret these marks and mediate downstream biological effects. BRD is the most well-characterized reader domain, while other recognition motifs include DPF and PH (Musselman et al., 2012) (See Table 3).

**TABLE 3:** Histone-acetylation enzymes.

Acetylation		
Writers	GNAT	HAT1 HAT4 GNC5
	p300/CBP	
	MYST	Tip60 MOZ MORF HBO1 MOF
Eraser	HDAC	
	Class I	HDAC1 HDAC2 HDAC3 HDAC8
	Class IIa	HDAC4 HDAC5 HDAC7 HDAC9
	Class IIb	HDAC6 HDAC10
	Class III	SIRT2 SIRT3 SIRT4 SIRT5 SIRT6 SIRT7
	Class IV	HDAC11
Readers	DPF PH BRD	

While the histone acetyltransferases are classified according to substrate recognition and cellular localization, histone deacetylases are classified into two groups according to the catalytic mechanisms (HDAC and SIRT). Likewise, the HDAC family has also been divided into four classes according to structural and functional characteristics. Abbreviations: HAT: Histone acetyltransferase; GNC5: GCN5-related N-acetyltransferases; p300/CBP: 300 kDa protein and CREB-binding protein. HDAC: histone deacetylases; SIRT: Sirtuins; BRD: bromodomains; DPF: double plant homeodomain finger; PH: double pleckstrin homology domain.

### 5.1 Role of histone modification in cardiac fibrosis

#### 5.1.1 Writers: histone modifications and cardiac fibrosis

Several studies have demonstrated the involvement of histone-modifying enzymes, or writers, in the regulation of CF. One such enzyme is DOT1L. Its expression has been directly correlated with elevated levels of profibrotic markers in CFBs. Notably, DOT1L regulates the transcription of forkhead box O3 (FOXO3), a transcription factor implicated in CFBs activation. Inhibition of DOT1L significantly reduces FOXO3 transcription, leading to a marked decrease in fibrogenic gene expression (Xu et al., 2022).

Another key writer implicated in CF is protein arginine methyltransferase 5 (PRMT5). Overexpression of PRMT5 in CFBs induces their activation and differentiation into myoFIBs, whereas its inhibition exerts the opposite effect. This process is mechanistically linked to the TGF-β1/Smad3 signaling pathway (Dong et al., 2023). *In vivo*, PRMT5 deficiency results in reduced CF, as evidenced by decreased expression of fibrotic markers such as α-SMA. In CFBs, PRMT5 inhibition downregulates Col1A1 and Acta2, and its interaction with Smad3 is essential for the recruitment of fibrogenic gene promoters (Katanasaka et al., 2024).

Further mechanistic insights reveal that the lysine methyltransferase complex WDR5/MLL1 (WD repeat-containing protein 5 and Mixed-Lineage Leukemia 1) recruited to PRMT5-dependent arginine methylation sites. This complex mediates H3K4me3, a modification associated with gene activation. Both PRMT5-induced arginine methylation and MLL1-induced lysine methylation cooperatively promote the transcription of α-SMA and myoFIBs differentiation. Disruption of WDR5/MLL1 leads to significant downregulation of Col1a1, Acta2, and α-SMA protein expression in CFBs (Katanasaka et al., 2024). Additionally, MLL1 has been implicated in cardiovascular inflammation by promoting the transcription of chemokines CCL3 and CCL4 through H3K4me3 modification, thereby contributing to the inflammatory response in atherosclerosis associated with diabetes (Chen et al., 2024).

The HAT p300 also plays a key role in CF. Silencing of p300 in TGF-β1-treated cellular models reduces the expression of collagen I and α-SMA while attenuating fibroblast activation. Moreover, p300 is involved in the acetylation and activation of Smad2, as its inhibition prevents Smad2 phosphorylation and nuclear localization (Lim et al., 2021). In a hypertensive mouse model, myocardial tissue exhibited increased differentiation of myoFIBs and H3K9 acetylation, correlating with elevated α-SMA expression. Pharmacological inhibition of p300 significantly reduced interstitial and perivascular collagen deposition in the myocardium (Rai et al., 2019).

#### 5.1.2 Erasers: histone modifications and cardiac fibrosis

Histone demethylases and deacetylases, collectively known as erasers, are critical regulators of epigenetic mechanisms associated with CF. Lysine demethylase 5B (KDM5B) has been identified as a key player in CF, as its expression increases under pathological stress conditions. Inhibition of KDM5B significantly reduces CF, as evidenced by decreased mRNA levels of fibrotic markers including Col1A1, Col3A1, fibronectin 1 (FN1), cell communication network factor 2 (CCN2), TGF-β, and Acta2 (Wang et al., 2022). Mechanistically, KDM5B exerts its profibrotic effects by binding to the promoter of activating transcription factor 3 (ATF3), a known antifibrotic regulator, suppressing its expression through H3K4me2/3 demethylation, thereby enhancing TGF-β signaling and profibrotic gene overexpression.

The role of KDM5B in cardiac remodeling has been further elucidated using TK-129, a potent pyrazole-based KDM5B inhibitor. TK-129 attenuates Ang II-induced CFBs activation *in vitro* and mitigates isoprenaline-induced myocardial remodeling and fibrosis *in vivo*. KDM5B upregulation has been associated with activation of the Wnt/β-catenin signaling pathway, suggesting an epigenetic link between KDM5B and this profibrotic signaling cascade (Tang et al., 2022).

In terms of histone deacetylation, HDAC8 has emerged as a key regulator in CF. The novel hydroxamic acid-based HDAC inhibitor YAK577 has been shown to attenuate left ventricular systolic dysfunction and CF in an isoproterenol (ISO)-induced heart failure model, partly by downregulating metalloproteinase 12 (MMP12). Both HDAC8 and CF-related genes (Col1A1, FN1) are upregulated in ISO-induced heart failure and TGF-β1-treated CFBs, whereas YAK577 treatment reverses these changes (Zhou et al., 2024). Furthermore, HDAC8 overexpression elevates MMP12 mRNA levels, while HDAC8 knockdown decreases its expression. Mechanistically, HDAC8 interacts with signal transducer and activator of transcription 3 (STAT3), facilitating its binding to the MMP12 promoter and potentially enhancing transcriptional activity.

#### 5.1.3 Readers: bromodomain-containing proteins and cardiac fibrosis

Epigenetic “reader” proteins recognize specific histone PTMs and play essential roles in the regulation of gene expression during CF. Among these, bromodomain-containing protein 4 (BRD4) has emerged as a critical regulator due to its ability to bind specifically to acetylated lysine residues on histone tails.

BRD4 has been identified as a promising therapeutic target in cardiac remodeling and heart failure. Inhibition of BRD4 using small-molecule inhibitors, such as JQ1, has been shown to reduce CF *in vivo* and prevent CFBs activation *in vitro* (He et al., 2022). These antifibrotic effects are mechanistically linked to modulation of the TGF-β1/Smad2/3 signaling pathway, which plays a central role in fibrotic gene expression. Additionally, BRD4 activity is modulated by the p38 mitogen-activated protein kinase (MAPK) pathway, further supporting its role in fibrotic remodeling (Stratton et al., 2019).

BRD4 inhibition has also been shown to counteract Ang II-induced myoFIBs differentiation, a process partly mediated by activation of the Keap1/Nrf2/HO-1 antioxidant pathway, highlighting a potential role in oxidative stress modulation (Han et al., 2023). Moreover, BRD4 has been implicated in atrial CF, where its silencing prevents ECM synthesis and CFBs activation (Song et al., 2024).

A recent innovative approach has introduced biomimetic nanoparticle-based drug delivery systems to enhance the specificity and efficacy of BRD4 inhibition in CF. One such system employs nanoparticles coated with platelet and erythrocyte membranes to facilitate targeted delivery of JQ1. The platelet membrane confers specificity for myoFIBs and cardiac collagen, while the erythrocyte membrane prolongs nanoparticle circulation time. This strategy has shown promising antifibrotic outcomes by effectively reducing CFBs activation and collagen secretion in myocardial tissue (Li Y. et al., 2024).

Histones are fundamental structural components of chromatin, and their functions are intricately regulated by a diverse array of PTMs. These modifications govern chromatin accessibility and transcriptional activity, ultimately shaping cellular responses such as CFBs activation and differentiation into myoFIBs. A comprehensive understanding of how chromatin-modifying enzymes including KMTs, PRMTs, KDMs, HATs, HDACs and their corresponding reader proteins orchestrate the transcriptional landscape in CF may provide valuable insights for the development of epigenetically targeted antifibrotic therapies.

## 6 DNA methylation

DNA methylation is a key epigenetic modification associated with gene silencing (Ping et al., 2014; Wu et al., 2014). This process regulates gene expression by recruiting transcriptional repressors and inhibiting transcription factor binding to DNA. During embryonic development, DNA methylation patterns are dynamically established and modified through *de novo* methylation and demethylation (Moore et al., 2013). DNA methylation primarily occurs at cytosines within CpG dinucleotides (Ikeda, 2023) and is catalyzed by DNA methyltransferases (DNMTs), including DNMT1, DNMT3A, and DNMT3B (Edwards et al., 2017). These modifications are recognized by protein families such as methyl-CpG-binding domain proteins and zinc finger proteins (e.g., Kaiso, ZBTB4, ZBTB38), which repress transcription by binding to methylated CpGs (Hendrich and Bird, 1998; Filion et al., 2006). Additionally, plant homeodomain (PHD) and Really Interesting New Gene (RING) finger domain-containing proteins help maintain DNA methylation by interacting with DNMT1, ensuring methylation pattern propagation during DNA replication (Deogharia and Gurha, 2024).

### 6.1 DNA methyltransferases (DNMT) as mediators of cardiac fibrosis

DNMTs play a crucial role in CF by regulating genes involved in cell differentiation and ECM production. α-SMA, a marker of CFBs-to-myoFIBs differentiation (Phan, 2002; Zhang et al., 2015), is modulated through promoter demethylation, which is facilitated by downregulation of DNMT1 (He et al., 2019).

Extracellular superoxide dismutase (EC-SOD) also influences DNA methylation under hypoxic stress by inhibiting DNMT1 and DNMT3B-mediated methylation of tumor suppressor genes such as RASSF1A, thereby reducing fibrotic markers (Rajgarhia et al., 2021).

DNMT1 overexpression suppresses the microRNA (miRNA) miR-152-3p, leading to activation of the Wnt/β-catenin signaling pathway. Conversely, DNMT1 inhibition restores miR-152-3p levels and reduces CFBs proliferation (Xu et al., 2021). In diabetic cardiomyopathy, DNMT1-mediated methylation of the suppressor of cytokine signaling 3 (SOCS3) promoter suppresses its expression, promoting STAT3 activation and fibrotic remodeling. Inhibition of DNMT1 reverses this effect (Tao et al., 2021).

Moreover, *in silico* analysis of heart failure patients with clonal hematopoiesis of indeterminate potential (CHIP) mutations in DNMT3A revealed enhanced monocyte-CFBs interactions, which were confirmed *in vivo* and associated with fibrotic remodeling (Shumliakivska et al., 2024).

DNMT3A also promotes CF via hypermethylation of the secreted frizzled-related protein 3 (sFRP3) promoter, leading to its repression. This downregulation impairs Drp1-regulated mitochondrial fission and promotes CFBs proliferation. DNMT3A knockdown restores sFRP3 expression, normalizes mitochondrial dynamics, and attenuates fibrosis (Jiang et al., 2024).

Collectively, DNMTs contribute significantly to CF through regulation of gene expression networks controlling CFBs differentiation, proliferation, and ECM deposition. Nevertheless, their precise and context-dependent roles require further investigation to inform the development of targeted epigenetic therapies.

## 7 Noncoding rna and cardiac fibrosis

Non-coding RNAs, including miRNAs, lncRNAs, and circular RNAs (circRNAs), regulate gene expression through diverse mechanisms such as miRNA sponging, direct interaction with messenger RNAs (mRNAs), protein scaffold formation, and even encoding regulatory peptides (Olson et al., 2024).

### 7.1 MicroRNAs (miRNAs) in cardiac fibrosis

MiRNAs are key post-transcriptional regulators that play a crucial role in CF by modulating gene expression related to CFBs activation, differentiation, and ECM production. For instance, microRNA-223 (miR-223) has been implicated in CF following myocardial infarction. Its overexpression enhances CFBs proliferation, migration, and fibrotic protein expression, whereas its inhibition yields opposite effects. Mechanistically, miR-223 targets and suppresses the RAS protein activator p21, leading to the activation of the RAS signaling pathway (Liu et al., 2018).

Similarly, miR-10a has been associated with prolonged atrial fibrillation and upregulation of profibrotic genes. Its silencing mitigates these pathological effects (Li et al., 2019). Another miRNA, miR-99b-3p, promotes CF by increasing the expression of FN1, COI I, vimentin, and α-SMA, and by stimulating CFBs proliferation and migration. This action is mediated via inhibition of glycogen synthase kinase-3 beta (GSK3β), leading to Smad3 activation (Yu et al., 2021). Conversely, miR-331 exerts antifibrotic effects by suppressing profibrotic genes through the TGF-β/Smad3 pathway. Its overexpression reduces the expression of Smad3 and Col1A1, both *in vitro* and *in vivo* fibrosis models (Yousefi et al., 2021).

miR-29a-3p has been identified as a modulator of CF. Knockdown of miR-29a-3p increases the expression of TGF-β, COI I and COI III in CFBs, reversing the antifibrotic effects of LE. LE and miR-29a-3p act synergistically to reduce CF (Wang et al., 2021). The tumor suppressor p53 has also been implicated in this mechanism. p53 directly binds to the promoter region of miR-29a-3p, upregulating its expression. LE promotes the expression of both p53 and miR-29a-3p, thereby inhibiting CFBs overactivation (Xi et al., 2023).

In addition, Xu et al. (2023) demonstrated that DNMT3A-mediated methylation of miR-145 inhibits autophagy, promoting CFBs proliferation and CF. miR-129-5p also contributes to fibrosis regulation by inhibiting CFBs-to-myoFIBs transition through the suppression of asporin and the transcription factor SOX9 (Medzikovic et al., 2023).

### 7.2 lncRNAs and circRNAs in cardiac fibrosis

LncRNAs and circRNAs are emerging as pivotal regulators in CF through their involvement in transcriptional and post-transcriptional gene regulation. Silencing of lncRNA NEAT1 has been shown to promote CF. This effect is mediated by DNMT3A-induced methylation of NEAT1, which activates the NLRP3 inflammasome pathway and triggers pyroptosis in CFBs, contributing to fibrotic progression (Ding et al., 2023b). Conversely, overexpression of lncRNA GAS5 exerts antifibrotic effects by negatively regulating NLRP3. This is achieved through reduced expression of caspase-1 and interleukin-1β (IL-1β). Moreover, GAS5 downregulates miR-217 and upregulates SIRT1 expression, collectively alleviating CF (Zhang et al., 2024).

With respect to circRNAs, the circNSD1/miR-429-3p axis plays a significant role in the modulation of CF. circNSD1 acts as a sponge for miR-429-3p, thereby increasing the expression of sulfatase 1 (SULF1) and activating the Wnt/β-catenin signaling pathway. This cascade enhances the fibrotic response in CFBs, as evidenced by increased expression of fibrotic proteins in response to circNSD1 overexpression (Ji et al., 2024).

## 8 Drugs targeting epigenetic modifications in cardiac fibrosis

Although no active clinical trials or approved drugs currently target epigenetic mechanisms specifically for the treatment of CF, preclinical studies have identified several epigenetic enzymes as potential therapeutic targets. Among these, the HAT p300 has received considerable attention due to its role in promoting the transcription of profibrotic genes via histone acetylation.

Curcumin, a natural compound with epigenetic properties, has been investigated as a selective inhibitor of p300. Its administration suppresses cardiomyocyte hypertrophy and fibrosis, accompanied by a reduction in brain natriuretic peptide (BNP) expression without affecting blood pressure. This effect appears to result from inhibition of p300 acetyltransferase activity, limiting the transcription of genes associated with oxidative stress and inflammation in the myocardium (Funamoto et al., 2022). Complementary studies have shown that curcumin also reduces perivascular fibrosis and downregulates pro-hypertrophic gene expression in hypertensive models, further supporting its potential as an epigenetic modulator with antifibrotic effects (Sunagawa et al., 2021).

Beyond histone modifications, RNA epigenetic regulation has also emerged as a relevant mechanism in CF, particularly through m6A methylation. In this context, metformin, widely used as a first-line therapy for type 2 diabetes mellitus, has demonstrated inhibitory effects on METTL3, a key methyltransferase responsible for m6A deposition. Although its role in CF remains under investigation, recent studies have shown that metformin downregulates METTL3 expression, potentially influencing mRNA stability and translation of transcripts involved in fibrotic pathways (Chen et al., 2023).

These epigenetic effects have been confirmed in both *in vitro* and *in vivo* models, where metformin mitigated fibrotic and inflammatory myocardial remodeling post-myocardial infarction (Loi et al., 2021), and attenuated diabetes-induced CF (Li et al., 2023). These findings support the potential of metformin as a candidate for drug repurposing in the epigenetic treatment of CF.

### 8.1 Clinical implications

This review highlights the critical role of epigenetic modifications in cardiac remodeling, emphasizing the function of “writers,” “erasers,” and “readers” in the regulation of CF. The evidence presented provides new insights into the therapeutic potential of targeting these epigenetic regulators.

The studies discussed herein demonstrate that epigenetic modifications significantly influence the cellular behavior of CFBs, positioning epigenetic interventions as promising strategies for the treatment of CF. The modulation of DNA methylation, histone modifications, and ncRNAs has shown potential in preclinical models to reverse fibrotic responses, offering novel therapeutic avenues for cardiac disease management.

Epigenetic therapies, including inhibitors of DNMTs and BRD4, as well as modulators of ncRNAs, are being explored as potential antifibrotic strategies. Some of these approaches, such as DNMT inhibitors (e.g., decitabine, 5-azacytidine) and BET protein inhibitors (e.g., JQ1, CPI-0610), have demonstrated efficacy in modulating fibrotic pathways in preclinical models, suggesting their potential translation into clinical applications.

Additionally, epigenetic biomarkers could serve as diagnostic and prognostic tools for CF. The identification of methylation patterns, histone modifications, and specific ncRNAs associated with fibrosis progression may enable earlier detection and risk stratification of patients. These biomarkers could also help monitor treatment response in future epigenetic-based therapies.

Despite these promising findings, several challenges remain before epigenetic therapies can be fully integrated into clinical practice. Given that epigenetic modifications regulate multiple cellular processes, ensuring treatment specificity and minimizing off-target effects will be critical. Future research should focus on optimizing targeted delivery systems, such as nanoparticle-based therapies, to enhance the selectivity of epigenetic drugs and reduce potential side effects.

By elucidating the molecular mechanisms underlying CF, this review identifies novel epigenetic targets that could be leveraged to modulate fibrosis progression, paving the way for innovative therapeutic approaches in cardiovascular disease. Further clinical and translational research is required to refine these strategies and determine their feasibility for human application.

## 9 Conclusion

Cardiac fibrosis (CF) is a prevalent pathophysiological process characterized by excessive ECM deposition, primarily driven by the activation and abnormal proliferation of cardiac fibroblasts (CFBs) into myofibroblasts (myoFIBs). These cells play a central role in ECM production and fibrotic remodeling, ultimately contributing to cardiac dysfunction (Garvin and Hale, 2022; Kong et al., 2014; Krenning et al., 2010).

**FIGURE 2:**
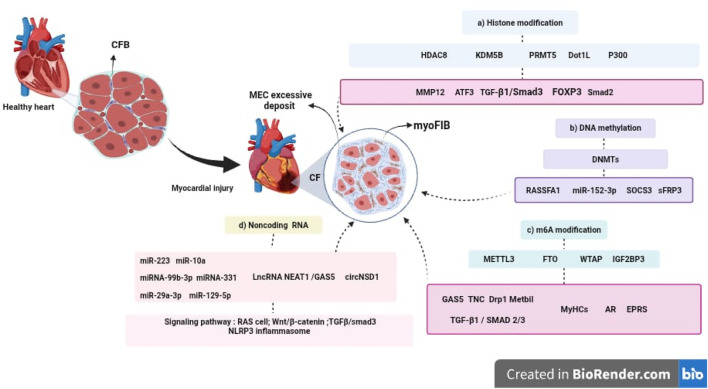
*Epigenetic mechanisms involved in the progression of CF.* Following myocardial injury, CFBs become activated and differentiate into myoFIBs, leading to excessive ECM deposition and the development of CF. This process is regulated by several epigenetic mechanisms, including: (a) Histone modifications, mediated by enzymes such as HDAC8, KDM5B, PRMT5, Dot1L, and p300, which influence the expression of key genes including *MMP12, TCF3, TGF-β1/Smad3, FOXP3*, and *Smad2*. (b) **DNA** methylation, regulated by DNA methyltransferases (DNMTs), affects genes such as *RASSF1, SOCS3, sFRP3*, and microRNAs like *miR-152-3p*. (c) m6A RNA modification, controlled by METTL3, FTO, WTAP, and IGF2BP3, impacts the expression of *GAS5, TNC, Drp1, Mettl1*, and *TGF-β1/SMAD2/3.* (d) Non-coding RNAs, including microRNAs (*miR-223, miR-10a, miR-99b-3p, miR-331, miR-29a-3p, miR-129-5p*), lncRNAs (*NEAT1, GAS5*), and circRNAs (*circNSD1*), which modulate signaling pathways involved in fibroblast activation and tissue remodeling, such as *RAS, Wnt/β-catenin, TGF-β/Smad3*, and *NLRP3 inflammasome*. These epigenetic regulators represent promising therapeutic targets for the development of innovative antifibrotic strategies in cardiovascular disease.

Epigenetic modifications, including DNA methylation, histone modifications, and ncRNAs, have emerged as key regulators of CF. The evidence reviewed suggests that epigenetic enzymes, referred to as “writers,” “readers,” and “erasers”, modulate fibrotic responses by influencing gene expression patterns, CFBs activation, and ECM synthesis. In particular, the m6A RNA modification machinery, DNMTs, and histone-modifying enzymes have been implicated in fibrosis progression, often through interactions with major signaling pathways such as Wnt/β-catenin and TGF-β/Smad (see Figure 2).

Despite recent advances, the precise role of specific epigenetic regulators in CF remains to be fully elucidated. While preclinical studies support the therapeutic potential of targeting DNMTs, BRD4, and ncRNAs, further research is needed to optimize selectivity, safety, and delivery strategies for epigenetic-based therapies.

### 9.1 Future prospects

To advance the clinical translation of epigenetic therapies, future research should focus on:

• Deciphering the interplay between different epigenetic mechanisms in CF pathophysiology, particularly in the regulation of CFBs-to-myoFIBs differentiation.
• Exploring the therapeutic potential of epigenetic inhibitors, such as DNMT and BRD4 inhibitors, and evaluating their effects in large-animal models and clinical trials.
• Developing targeted delivery systems, such as nanoparticle-based epigenetic therapies, to enhance specificity and minimize off-target effects.
• Identifying and validating epigenetic biomarkers for CF, which could aid in early diagnosis, prognosis, and monitoring of therapeutic responses.
• Investigating antifibrotic factors that inhibit CFBs-to-myoFIBs transition, as these may offer complementary or synergistic effects with epigenetic interventions.

Understanding the intricate regulatory networks of epigenetic modifications in CF will pave the way for novel therapeutic strategies in cardiovascular disease, ultimately improving patient outcomes and addressing the unmet clinical need for effective antifibrotic therapies.

## Glossary

CF: Cardiac fibrosis
ECM: extracellular matrix
myoFIB: myofibroblasts
m6A: N6-methyladenosine
METTL3: methyltransferase 3
METTL14: methyltransferase 14
WTAP: Wilms tumor-associated protein 1
SAM: S-adenosylmethionine
IGF2BP: insulin-like growth factor 2 mRNA binding proteins
FTO: alpha-ketoglutarate-dependent dioxygenase
TGF-β1: transforming growth factor beta 1
AR: Androgen receptor
Drp1: dynamin-related protein 1
LE: leonurine
KMTs: histone methyltransferases acting on lysines
PRMT: arginine N-methyltransferase
KDMs: Lysine demethylases
HDACs: histone deacetylases
GNATs: GCN5-related N-acetyltransferases
p300: 300 kDa protein
CBP: CREB-binding protein
SIRTs: sirtuins
BAH: bromo-adjacent homology
DCD: chromobarrel, chromodomain, double chromodomain
MBT: malignant brain tumor
TTD: Tudor tandem domain
Zf-CW: zinc finger CW
DOT1: DOT1-like histone lysine methyltransferase
Col1a1: alpha 1 chain
H3K4: trimethylates histone H3
MMP12: metalloproteinase 12
DNMTs: DNA methyltransferases
SOCS3: Cytokine signaling 3
sFRP3: frizzled-related protein 3
ncRNA: non-coding RNA

## References

[B1] Aguado-Alvaro L. P. Garitano N. Pelacho B. (2024). Fibroblast diversity and epigenetic regulation in cardiac fibrosis. Int. J. Mol. Sci. 25 (11), 6004. 10.3390/ijms25116004 38892192 PMC11172550

[B2] Black J. C. Van Rechem C. Whetstine J. R. (2012). Histone lysine methylation dynamics: establishment, regulation, and biological impact. Mol. cell 48 (4), 491–507. 10.1016/j.molcel.2012.11.006 23200123 PMC3861058

[B3] Cao Y. Lu L. Liu M. Li X. C. Sun R. R. Zheng Y. (2014). Impact of epigenetics in the management of cardiovascular disease: a review. Eur. Rev. Med. Pharmacol. Sci. 18 (20), 3097–3104. 25392111

[B4] Chen C. J. Huang J. Y. Huang J. Q. Deng J. Y. Shangguan X. H. Chen A. Z. (2023). Metformin attenuates multiple myeloma cell proliferation and encourages apoptosis by suppressing METTL3-mediated m6A methylation of THRAP3, RBM25, and USP4. Cell. Cycle 22, 986–1004. 10.1080/15384101.2023.2170521 36762777 PMC10054227

[B5] Chen J. Jamaiyar A. Wu W. Hu Y. Zhuang R. Sausen G. (2024). Deficiency of lncRNA MERRICAL abrogates macrophage chemotaxis and diabetes-associated atherosclerosis. Cell Rep. 43 (3), 113815. 10.1016/j.celrep.2024.113815 38428421 PMC11006532

[B6] Cheng H. Li L. Xue J. Ma J. Ge J. (2023). TNC accelerates hypoxia-induced cardiac injury in a METTL3-dependent manner. Genes 14 (3), 591. 10.3390/genes14030591 36980863 PMC10048594

[B7] Delacroix C. Hulot J. S. (2022). Rôle des intégrines dans la fibrose cardiaque. Med. Sci. M/S 38 (5), 438–444. 10.1051/medsci/2022055 35608466

[B8] Deogharia M. Gurha P. (2024). Epigenetic regulation of heart failure. Curr. Opin. Cardiol. 39 (4), 371–379. 10.1097/HCO.0000000000001150 38606626 PMC11150090

[B9] Díaz-Araya G. Vivar R. Humeres C. Boza P. Bolivar S. Muñoz C. (2015). Cardiac fibroblasts as sentinel cells in cardiac tissue: receptors, signaling pathways and cellular functions. Pharmacol. Res. 101, 30–40. 10.1016/j.phrs.2015.07.001 26151416

[B10] Ding J. F. Sun H. Song K. Zhou Y. Tu B. Shi K. H. (2023a). IGFBP3 epigenetic promotion induced by METTL3 boosts cardiac fibroblast activation and fibrosis. Eur. J. Pharmacol. 942, 175494. 10.1016/j.ejphar.2023.175494 36657656

[B11] Ding J. F. Zhou Y. Xu S. S. Shi K. H. Sun H. Tu B. (2023b). Epigenetic control of LncRNA NEAT1 enables cardiac fibroblast pyroptosis and cardiac fibrosis. Eur. J. Pharmacol. 938, 175398. 10.1016/j.ejphar.2022.175398 36455647

[B12] Dong X. L. Yuan B. H. Yu S. Z. Liu H. Pan X. H. Sun J. (2023). Adriamycin induces cardiac fibrosis in mice via PRMT5-mediated cardiac fibroblast activation. Acta Pharmacol. Sin. 44 (3), 573–583. 10.1038/s41401-022-00963-x 36056082 PMC9958096

[B13] Edwards J. R. Yarychkivska O. Boulard M. Bestor T. H. (2017). DNA methylation and DNA methyltransferases. Epigenetics and chromatin 10, 23. 10.1186/s13072-017-0130-8 28503201 PMC5422929

[B14] Esteller M. (2017). Epigenetic drugs: more than meets the eye. Epigenetics 12 (5), 307. 10.1080/15592294.2017.1322881 28541128 PMC5453189

[B15] Fellows R. Varga-Weisz P. (2020). Chromatin dynamics and histone modifications in intestinal microbiota-host crosstalk. Mol. Metab. 38, 100925. 10.1016/j.molmet.2019.12.005 31992511 PMC7300386

[B16] Filion G. J. Zhenilo S. Salozhin S. Yamada D. Prokhortchouk E. Defossez P. A. (2006). A family of human zinc finger proteins that bind methylated DNA and repress transcription. Mol. Cell. Biol. 26 (1), 169–181. 10.1128/MCB.26.1.169-181.2006 16354688 PMC1317629

[B17] Frangogiannis N. (2020). Transforming growth factor-β in tissue fibrosis. J. Exp. Med. 217 (3), e20190103. 10.1084/jem.20190103 32997468 PMC7062524

[B18] Funamoto M. Sunagawa Y. Katanasaka Y. Kato T. Funada J. Ajiro Y. (2022). Effects of high-absorption curcumin for the prevention of hypertensive heart disease: a double-blind, placebo-controlled, randomized clinical study. Eur. heart J. open 2 (5), oeac057. 10.1093/ehjopen/oeac057 36172003 PMC9512148

[B19] Gao Y. Guo Q. Yu L. (2024). m6A modification of RNA in cervical cancer: role and clinical perspectives. RNA Biol. 21 (1), 49–61. 10.1080/15476286.2024.2408707 39344658 PMC11445900

[B20] Garvin A. M. Hale T. M. (2022). State of change: epigenetic and mitochondrial regulation of cardiac fibroblast activation. Curr. Opin. Physiol. 28 (28), 100557. 10.1016/j.cophys.2022.100557

[B21] Gong F. Miller K. M. (2019). Histone methylation and the DNA damage response. Rev. Mutat. Res. 780, 37–47. 10.1016/j.mrrev.2017.09.003 PMC6690396 31395347

[B22] Guo P. Chen W. Li H. Li M. Li L. (2018). The histone acetylation modifications of breast cancer and their therapeutic implications. Pathology Oncol. Res. POR 24 (4), 807–813. 10.1007/s12253-018-0433-5 29948617

[B23] Han J. Zhang Y. Peng H. (2023). Fucoxanthin inhibits cardiac fibroblast transdifferentiation by alleviating oxidative stress through downregulation of BRD4. PloS one 18 (9), e0291469. 10.1371/journal.pone.0291469 37699016 PMC10497131

[B24] He Y. Ling S. Sun Y. Sheng Z. Chen Z. Pan X. (2019). DNA methylation regulates α-smooth muscle actin expression during cardiac fibroblast differentiation. J. Cell Physiol. 234 (5), 7174–7185. 10.1002/jcp.27471 30362530

[B25] He Z. Jiao H. An Q. Zhang X. Zengyangzong D. Xu J. (2022). Discovery of novel 4-phenylquinazoline-based BRD4 inhibitors for cardiac fibrosis. Acta Pharm. Sin. B 12 (1), 291–307. 10.1016/j.apsb.2021.07.018 35127386 PMC8799877

[B26] Hendrich B. Bird A. (1998). Identification and characterization of a family of mammalian methyl-CpG binding proteins. Mol. Cell. Biol. 18 (11), 6538–6547. 10.1128/MCB.18.11.6538 9774669 PMC109239

[B27] Heymans S. González A. Pizard A. Papageorgiou A. P. López-Andrés N. Jaisser F. (2015). Searching for new mechanisms of myocardial fibrosis with diagnostic and/or therapeutic potential. Eur. J. heart Fail. 17 (8), 764–771. 10.1002/ejhf.312 26126780

[B28] Hu T. Wang G. Wang D. Deng Y. Wang W. (2025). m6A methylation modification: potential pathways to suppress osteosarcoma metastasis. Int. Immunopharmacol. 145, 113759. 10.1016/j.intimp.2024.113759 39662272

[B29] Huang B. Xie L. Ke M. Fan Y. Tan J. Ran J. (2023). Programmed release METTL3-14 inhibitor microneedle protects myocardial function by reducing Drp1 m6A modification-mediated mitochondrial fission. ACS Appl. Mater. and interfaces 15 (40), 46583–46597. 10.1021/acsami.3c06318 37752784 PMC10573327

[B30] Ikeda S. (2023). Current status of genome-wide epigenetic profiling of mammalian preimplantation embryos. Reproductive Med. Biol. 22 (1), e12521. 10.1002/rmb2.12521 PMC10283350 37351110

[B31] Izzo A. Schneider R. (2010). Chatting histone modifications in mammals. Briefings Funct. genomics 9 (5-6), 429–443. 10.1093/bfgp/elq024 PMC3080777 21266346

[B32] Ji D. N. Jin S. D. Jiang Y. Xu F. Y. Fan S. W. Zhao Y. L. (2024). CircNSD1 promotes cardiac fibrosis through targeting the miR-429-3p/SULF1/Wnt/β-catenin signaling pathway. Acta Pharmacol. Sin. 45 (10), 2092–2106. 10.1038/s41401-024-01296-7 38760544 PMC11420342

[B33] Jiang S. X. Zhou Z. Y. Tu B. Song K. Lin L. C. Liu Z. Y. (2024). Epigenetic regulation of mitochondrial fission and cardiac fibrosis via sFRP3 promoter methylation. Cell. Mol. life Sci. CMLS 81 (1), 483. 10.1007/s00018-024-05516-5 39644393 PMC11625034

[B34] Katanasaka Y. Yabe H. Murata N. Sobukawa M. Sugiyama Y. Sato H. (2024). Fibroblast-specific PRMT5 deficiency suppresses cardiac fibrosis and left ventricular dysfunction in male mice. Nat. Commun. 15 (1), 2472. 10.1038/s41467-024-46711-z 38503742 PMC10951424

[B35] Kong P. Christia P. Frangogiannis N. G. (2014). The pathogenesis of cardiac fibrosis. Cell. Mol. Life Sci. 71 (71), 549–574. 10.1007/s00018-013-1349-6 23649149 PMC3769482

[B36] Krenning G. Zeisberg E. M. Kalluri R. (2010). The origin of fibroblasts and mechanism of cardiac fibrosis. J. Cell. Physiol. 225, 631–637. 10.1002/jcp.22322 20635395 PMC3098503

[B37] Lee J. M. Hammarén H. M. Savitski M. M. Baek S. H. (2023). Control of protein stability by post-translational modifications. Nat. Commun. 14 (1), 201. 10.1038/s41467-023-35795-8 36639369 PMC9839724

[B38] Li J. Wei L. Hu K. He Y. Gong G. Liu Q. (2024a). Deciphering m6A methylation in monocyte-mediated cardiac fibrosis and monocyte-hitchhiked erythrocyte microvesicle biohybrid therapy. Theranostics 14 (9), 3486–3508. 10.7150/thno.95664 38948064 PMC11209724

[B39] Li P. F. He R. H. Shi S. B. Li R. Wang Q. T. Rao G. T. (2019). Modulation of miR-10a-mediated TGF-β1/Smads signaling affects atrial fibrillation-induced cardiac fibrosis and cardiac fibroblast proliferation. Biosci. Rep. 39 (2), BSR20181931. 10.1042/BSR20181931 30683806 PMC6367129

[B40] Li T. Zhuang Y. Yang W. Xie Y. Shang W. Su S. (2021). Silencing of METTL3 attenuates cardiac fibrosis induced by myocardial infarction via inhibiting the activation of cardiac fibroblasts. FASEB J. official Publ. Fed. Am. Soc. Exp. Biol. 35 (2), e21162. 10.1096/fj.201903169R 33150686

[B41] Li Y. Liu X. Wan L. Han B. Ma S. Pan H. (2023). Metformin suppresses cardiac fibroblast proliferation under high-glucose conditions via regulating the mitochondrial complex I protein Grim-19 involved in the Sirt1/Stat3 signaling pathway. Free Radic. Biol. Med. 206, 1–12. 10.1016/j.freeradbiomed.2023.06.013 37353174

[B42] Li Y. Yu J. Cheng C. Chen W. Lin R. Wang Y. (2024b). Platelet and erythrocyte membranes coassembled biomimetic nanoparticles for heart failure treatment. ACS nano 18 (39), 26614–26630. 10.1021/acsnano.4c04814 39174015 PMC11447889

[B43] Lim Y. Jeong A. Kwon D. H. Lee Y. U. Kim Y. K. Ahn Y. (2021). P300/CBP-Associated factor activates cardiac fibroblasts by SMAD2 acetylation. Int. J. Mol. Sci. 22 (18), 9944. 10.3390/ijms22189944 34576109 PMC8472677

[B44] Ling C. Rönn T. (2019). Epigenetics in human obesity and type 2 diabetes. Cell metab. 29 (5), 1028–1044. 10.1016/j.cmet.2019.03.009 30982733 PMC6509280

[B45] Liu M. López de Juan Abad B. Cheng K. (2021). Cardiac fibrosis: myofibroblast-mediated pathological regulation and drug delivery strategies. Adv. drug Deliv. Rev. 173, 504–519. 10.1016/j.addr.2021.03.021 33831476 PMC8299409

[B46] Liu X. Xu Y. Deng Y. Li H. (2018). MicroRNA-223 regulates cardiac fibrosis after myocardial infarction by targeting RASA1. Cell. physiology Biochem. Int. J. Exp. Cell. physiology, Biochem. Pharmacol. 46 (4), 1439–1454. 10.1159/000489185 29689569

[B47] Liu Z. Y. Lin L. C. Liu Z. Y. Song K. Tu B. Sun H. (2024). N6-Methyladenosine-mediated phase separation suppresses NOTCH1 expression and promotes mitochondrial fission in diabetic cardiac fibrosis. Cardiovasc. Diabetol. 23 (1), 347. 10.1186/s12933-024-02444-3 39342271 PMC11439301

[B48] Liu Z. Y. Song K. Tu B. Lin L. C. Sun H. Zhou Y. (2023). Crosstalk between oxidative stress and epigenetic marks: new roles and therapeutic implications in cardiac fibrosis. Redox Biol. 65, 102820. 10.1016/j.redox.2023.102820 37482041 PMC10369469

[B49] Loi H. Kramar S. Laborde C. Marsal D. Pizzinat N. Cussac D. (2021). Metformin attenuates postinfarction myocardial fibrosis and inflammation in mice. Int. J. Mol. Sci. 22 (17), 9393. 10.3390/ijms22179393 34502314 PMC8430638

[B50] Loscalzo J. Handy D. E. (2014). Epigenetic modifications: basic mechanisms and role in cardiovascular disease (2013 Grover Conference series). Pulm. Circ. 4 (2), 169–174. 10.1086/675979 25006435 PMC4070783

[B51] Luo J. Liu H. Luan S. He C. Li Z. (2018). Aberrant regulation of mRNA m^6^A modification in cancer development. Int. J. Mol. Sci. 19 (9), 2515. 10.3390/ijms19092515 30149601 PMC6164065

[B52] Ma L. Chang X. Gao J. Zhang Y. Chen Y. Zhou H. (2024). METTL3 boosts mitochondrial fission and induces cardiac fibrosis after ischemia/reperfusion injury. Int. J. Biol. Sci. 20 (2), 433–445. 10.7150/ijbs.87535 38169612 PMC10758110

[B53] Mathiyalagan P. Adamiak M. Mayourian J. Sassi Y. Liang Y. Agarwal N. (2019). FTO-dependent N6-methyladenosine regulates cardiac function during remodeling and repair. Circulation 139 (4), 518–532. 10.1161/CIRCULATIONAHA.118.033794 29997116 PMC6400591

[B54] Medzikovic L. Aryan L. Ruffenach G. Li M. Savalli N. Sun W. (2023). Myocardial fibrosis and calcification are attenuated by microRNA-129-5p targeting Asporin and Sox9 in cardiac fibroblasts. JCI insight 8 (9), e168655. 10.1172/jci.insight.168655 37154157 PMC10243800

[B55] Meng Y. Xi T. Fan J. Yang Q. Ouyang J. Yang J. (2024). The inhibition of FTO attenuates the antifibrotic effect of leonurine in rat cardiac fibroblasts. Biochem. biophysical Res. Commun. 693, 149375. 10.1016/j.bbrc.2023.149375 38128243

[B56] Miller J. L. Grant P. A. (2013). The role of DNA methylation and histone modifications in transcriptional regulation in humans. Sub-cellular Biochem. 61, 289–317. 10.1007/978-94-007-4525-4_13 PMC6611551 23150256

[B57] Miziak P. Baran M. Borkiewicz L. Trombik T. Stepulak A. (2024). Acetylation of histone H3 in cancer progression and prognosis. Int. J. Mol. Sci. 25 (20), 10982. 10.3390/ijms252010982 39456765 PMC11507103

[B58] Moore L. D. Le T. Fan G. (2013). DNA methylation and its basic function. Neuropsychopharmacology 38 (1), 23–38. 10.1038/npp.2012.112 22781841 PMC3521964

[B59] Musselman C. A. Lalonde M. E. Côté J. Kutateladze T. G. (2012). Perceiving the epigenetic landscape through histone readers. Nat. Struct. and Mol. Biol. 19 (12), 1218–1227. 10.1038/nsmb.2436 23211769 PMC3645987

[B60] Oerum S. Meynier V. Catala M. Tisné C. (2021). A comprehensive review of m6A/m6Am RNA methyltransferase structures. Nucleic acids Res. 49 (13), 7239–7255. 10.1093/nar/gkab378 34023900 PMC8287941

[B61] Olson S. R. Tang W. H. W. Liu C. F. (2024). Non-coding ribonucleic acids as diagnostic and therapeutic targets in cardiac fibrosis. Curr. heart Fail. Rep. 21 (3), 262–275. 10.1007/s11897-024-00653-1 38485860 PMC11090942

[B62] Orsolic I. Carrier A. Esteller M. (2023). Genetic and epigenetic defects of the RNA modification machinery in cancer. Trends Genet. TIG 39 (1), 74–88. 10.1016/j.tig.2022.10.004 36379743

[B63] Park S. Y. Kim J. S. (2020). A short guide to histone deacetylases including recent progress on class II enzymes. Exp. and Mol. Med. 52 (2), 204–212. 10.1038/s12276-020-0382-4 32071378 PMC7062823

[B64] Peterson C. L. Laniel M. A. (2004). Histones and histone modifications. Curr. Biol. CB 14 (14), R546–R551. 10.1016/j.cub.2004.07.007 15268870

[B65] Phan S. H. (2002). The myofibroblast in pulmonary fibrosis. Chest 122 (6), 286S–289S. 10.1378/chest.122.6_suppl.286s 12475801

[B66] Ping J. Wang J. F. Liu L. Yan Y. E. Liu F. Lei Y. Y. (2014). Prenatal caffeine ingestion induces aberrant DNA methylation and histone acetylation of steroidogenic factor 1 and inhibits fetal adrenal steroidogenesis. Toxicology 321, 53–61. 10.1016/j.tox.2014.03.011 24717552

[B67] Plikus M. V. Wang X. Sinha S. Forte E. Thompson S. M. Herzog E. L. (2021). Fibroblasts: origins, definitions, and functions in health and disease. Cell 184 (15), 3852–3872. 10.1016/j.cell.2021.06.024 34297930 PMC8566693

[B68] Poziello A. Nebbioso A. Stunnenberg H. G. Martens J. H. A. Carafa V. Altucci L. (2021). Recent insights into Histone Acetyltransferase-1: biological function and involvement in pathogenesis. Epigenetics 16 (8), 838–850. 10.1080/15592294.2020.1827723 33016232 PMC8330999

[B69] Rai R. Sun T. Ramirez V. Lux E. Eren M. Vaughan D. E. (2019). Acetyltransferase p300 inhibitor reverses hypertension-induced cardiac fibrosis. J. Cell. Mol. Med. 23 (4), 3026–3031. 10.1111/jcmm.14162 30710427 PMC6433695

[B70] Rajgarhia A. Ayasolla K. R. Zaghloul N. Lopez Da Re J. M. Miller E. J. Ahmed M. (2021). Extracellular superoxide dismutase (EC-SOD) regulates gene methylation and cardiac fibrosis during chronic hypoxic stress. Front. Cardiovasc. Med. 8, 669975. 10.3389/fcvm.2021.669975 34136546 PMC8202000

[B71] Rothbart S. B. Strahl B. D. (2014). Interpreting the language of histone and DNA modifications. Biochimica biophysica acta 1839 (8), 627–643. 10.1016/j.bbagrm.2014.03.001 PMC4099259 24631868

[B72] Shumliakivska M. Luxán G. Hemmerling I. Scheller M. Li X. Müller-Tidow C. (2024). DNMT3A clonal hematopoiesis-driver mutations induce cardiac fibrosis by paracrine activation of fibroblasts. Nat. Commun. 15 (1), 606. 10.1038/s41467-023-43003-w 38242884 PMC10799021

[B73] Song K. Sun H. Tu B. Zhou Y. Lin L. C. Liu Z. Y. (2023). WTAP boosts lipid oxidation and induces diabetic cardiac fibrosis by enhancing AR methylation. iScience 26 (10), 107931. 10.1016/j.isci.2023.107931 37810250 PMC10558737

[B74] Song S. Yuan J. Fang G. Li Y. Ding S. Wang Y. (2024). BRD4 as a therapeutic target for atrial fibrosis and atrial fibrillation. Eur. J. Pharmacol. 977, 176714. 10.1016/j.ejphar.2024.176714 38849043

[B75] Stratton M. S. Bagchi R. A. Felisbino M. B. Hirsch R. A. Smith H. E. Riching A. S. (2019). Dynamic chromatin targeting of BRD4 stimulates cardiac fibroblast activation. Circulation Res. 125 (7), 662–677. 10.1161/CIRCRESAHA.119.315125 31409188 PMC7310347

[B76] Sun L. Zhang H. Gao P. (2022). Metabolic reprogramming and epigenetic modifications on the path to cancer. Protein and cell 13 (12), 877–919. 10.1007/s13238-021-00846-7 34050894 PMC9243210

[B77] Sun T. Wu R. Ming L. (2019). The role of m6A RNA methylation in cancer. Biomed. and Pharmacother. = Biomedecine and Pharmacother. 112, 108613. 10.1016/j.biopha.2019.108613 30784918

[B78] Sunagawa Y. Funamoto M. Shimizu K. Shimizu S. Sari N. Katanasaka Y. (2021). Curcumin, an inhibitor of p300-HAT activity, suppresses the development of hypertension-induced left ventricular hypertrophy with preserved ejection fraction in dahl rats. Nutrients 13 (8), 2608. 10.3390/nu13082608 34444769 PMC8397934

[B79] Tallquist M. D. (2020). Cardiac fibroblast diversity. Annu. Rev. physiology 82, 63–78. 10.1146/annurev-physiol-021119-034527 PMC10939057 32040933

[B80] Tan M. Liu S. Liu L. (2024). N6-methyladenosine (m6A) RNA modification in fibrosis and collagen-related diseases. Clin. epigenetics 16 (1), 127. 10.1186/s13148-024-01736-5 39261973 PMC11391634

[B81] Tang K. Jiao L. M. Qi Y. R. Wang T. C. Li Y. L. Xu J. L. (2022). Discovery of novel pyrazole-based KDM5B inhibitor TK-129 and its protective effects on myocardial remodeling and fibrosis. J. Med. Chem. 65 (19), 12979–13000. 10.1021/acs.jmedchem.2c00797 36112701

[B82] Tao H. Shi P. Zhao X. D. Xuan H. Y. Gong W. H. Ding X. S. (2021). DNMT1 deregulation of SOCS3 axis drives cardiac fibroblast activation in diabetic cardiac fibrosis. J. Cell. physiology 236 (5), 3481–3494. 10.1002/jcp.30078 32989761

[B83] Tsai Y. C. Hsieh T. H. Liao Y. R. Tsai M. T. Lin T. P. Lee D. Y. (2024). METTL3-Mediated N 6 -methyladenosine mRNA modification and cGAS-STING pathway activity in kidney fibrosis. J. Am. Soc. Nephrol. JASN 35 (10), 1312–1329. 10.1681/ASN.0000000000000428 39352860 PMC11452136

[B84] Tu B. Song K. Zhou Y. Sun H. Liu Z. Y. Lin L. C. (2023). METTL3 boosts mitochondrial fission and induces cardiac fibrosis by enhancing LncRNA GAS5 methylation. Pharmacol. Res. 194, 106840. 10.1016/j.phrs.2023.106840 37379961

[B85] Wang B. Tan Y. Zhang Y. Zhang S. Duan X. Jiang Y. (2022). Loss of KDM5B ameliorates pathological cardiac fibrosis and dysfunction by epigenetically enhancing ATF3 expression. Exp. and Mol. Med. 54 (12), 2175–2187. 10.1038/s12276-022-00904-y 36481938 PMC9794816

[B86] Wang J. Li Y. Deng L. Zha Y. Zhang S. (2024). FTO suppresses cardiac fibrosis after myocardial infarction via m6A-mediated epigenetic modification of EPRS. Mol. Med. Camb. Mass. 30 (1), 213. 10.1186/s10020-024-0098 39538146 PMC11562098

[B87] Wang R. Peng L. Lv D. Shang F. Yan J. Li G. (2021). Leonurine attenuates myocardial fibrosis through upregulation of miR-29a-3p in mice post-myocardial infarction. J. Cardiovasc. Pharmacol. 77 (2), 189–199. 10.1097/FJC.0000000000000957 33235025

[B88] Wang T. Kong S. Tao M. Ju S. (2020). The potential role of RNA N6-methyladenosine in Cancer progression. Mol. cancer 19 (1), 88. 10.1186/s12943-020-01204-7 32398132 PMC7216508

[B89] Wu J. Salva K. A. Stutz N. Jack L. B. Spiegelman V. S. Wood G. S. (2014). Quantitative gene analysis of methylation and expression (Q-GAME) in fresh or fixed cells and tissues. Exp. Dermatol. 23, 304–309. 10.1111/exd.12374 24646432 PMC4331060

[B90] Xi T. Wang R. Pi D. Ouyang J. Yang J. (2023). The p53/miR-29a-3p axis mediates the antifibrotic effect of leonurine on angiotensin II-stimulated rat cardiac fibroblasts. Exp. cell Res. 426 (1), 113556. 10.1016/j.yexcr.2023.113556 36933858

[B91] Xiao T. Wang P. Wu M. Cheng C. Yang Y. Bian Q. (2024). METTL3-regulated m6A modification of lncRNA E230001N04Rik is involved in myofibroblast differentiation in arsenic-induced pulmonary fibrosis through promoting senescence of lung epithelial cells. J. Hazard. Mater. 480, 136094. 10.1016/j.jhazmat.2024.136094 39405678

[B92] Xu J. Wang J. Long F. Zhong W. Su H. Su Z. (2022). Inhibition of the cardiac fibroblast-enriched histone methyltransferase Dot1L prevents cardiac fibrosis and cardiac dysfunction. Cell and Biosci. 12 (1), 134. 10.1186/s13578-022-00877-5 PMC9392317 35986422

[B93] Xu S. Zhang Y. Zhou G. Liu A. (2023). Bidirectional negative feedback actions of DNMT3A and miR-145 in regulating autophagy in cardiac fibroblasts and affecting myocardial fibrosis. J. bioenergetics Biomembr. 55 (5), 341–352. 10.1007/s10863-023-09980-9 37610521

[B94] Xu S. S. Ding J. F. Shi P. Shi K. H. Tao H. (2021). DNMT1-Induced miR-152-3p suppression facilitates cardiac fibroblast activation in cardiac fibrosis. Cardiovasc. Toxicol. 21 (12), 984–999. 10.1007/s12012-021-09690-x 34424481

[B95] Yao W. Hu X. Wang X. (2024). Crossing epigenetic frontiers: the intersection of novel histone modifications and diseases. Signal Transduct. Target. Ther. 9 (1), 232. 10.1038/s41392-024-01918-w 39278916 PMC11403012

[B96] Ye W. Lv X. Gao S. Li Y. Luan J. Wang S. (2023). Emerging role of m6A modification in fibrotic diseases and its potential therapeutic effect. Biochem. Pharmacol. 218, 115873. 10.1016/j.bcp.2023.115873 37884198

[B97] Yousefi F. Soltani B. M. Rabbani S. (2021). MicroRNA-331 inhibits isoproterenol-induced expression of profibrotic genes in cardiac myofibroblasts via the TGFβ/smad3 signaling pathway. Sci. Rep. 11 (1), 2548. 10.1038/s41598-021-82226-z 33510328 PMC7843612

[B98] Yu Y. H. Zhang Y. H. Ding Y. Q. Bi X. Y. Yuan J. Zhou H. (2021). MicroRNA-99b-3p promotes angiotensin II-induced cardiac fibrosis in mice by targeting GSK-3β. Acta Pharmacol. Sin. 42 (5), 715–725. 10.1038/s41401-020-0498-z 32814818 PMC8115164

[B99] Zhang C. Zhu Y. Zhang Y. Gao L. Zhang N. Feng H. (2015). Therapeutic potential of umbilical cord mesenchymal stem cells for inhibiting myofibroblastic differentiation of irradiated human lung fibroblasts. Tohoku J. Exp. Med. 236 (3), 209–217. 10.1620/tjem.236.209 26105694

[B100] Zhang Y. H. Sun T. T. Liu Z. H. Li X. Fan X. F. Han L. P. (2024). LncRNA GAS5 restrains ISO-induced cardiac fibrosis by modulating mir-217 regulation of SIRT1. Sci. Rep. 14 (1), 7652. 10.1038/s41598-024-58239-9 38561456 PMC10985102

[B101] Zhao B. S. Roundtree I. A. He C. (2017). Post-transcriptional gene regulation by mRNA modifications. Nat. Rev. Mol. cell Biol. 18 (1), 31–42. 10.1038/nrm.2016.132 27808276 PMC5167638

[B102] Zhou H. Kee H. J. Wan L. Asfaha Y. Fischer F. Kassack M. U. (2024). YAK577 attenuates cardiac remodeling and fibrosis in isoproterenol-infused heart failure mice by downregulating MMP12. Korean circulation J. 55, 231–247. 10.4070/kcj.2024.0093 39601396 PMC11922594

[B103] Zhou Y. Song K. Tu B. Sun H. Ding J. F. Luo Y. (2022). METTL3 boosts glycolysis and cardiac fibroblast proliferation by increasing AR methylation. Int. J. Biol. Macromol. 223 (Pt A), 899–915. 10.1016/j.ijbiomac.2022.11.042 36370857

[B104] Zhu D. H. Su K. K. Ou-Yang X. X. Zhang Y. H. Yu X. P. Li Z. H. (2024). Mechanisms and clinical landscape of N6-methyladenosine (m6A) RNA modification in gastrointestinal tract cancers. Mol. Cell. Biochem. 479 (7), 1553–1570. 10.1007/s11010-024-05040-x 38856795 PMC11254988

[B105] Zhuang Y. Li T. Hu X. Xie Y. Pei X. Wang C. (2023). MetBil as a novel molecular regulator in ischemia-induced cardiac fibrosis via METTL3-mediated m6A modification. FASEB J. official Publ. Fed. Am. Soc. Exp. Biol. 37 (3), e22797. 10.1096/fj.202201734R 36753405

